# Novel G10P[14] Rotavirus Strain, Northern Territory, Australia

**DOI:** 10.3201/eid.1908.121653

**Published:** 2013-08

**Authors:** Daniel Cowley, Celeste M. Donato, Susie Roczo-Farkas, Carl D. Kirkwood

**Affiliations:** Murdoch Childrens Research Institute, Melbourne, Victoria, Australia (D. Cowley, C.M. Donato, S. Roczo-Farkas, C.D. Kirkwood);; La Trobe University, Melbourne (C.M. Donato, C.D. Kirkwood)

**Keywords:** Rotavirus, G10P[14], group A rotavirus full genome analysis, Australia, enteric infections, indigenous Australian, viruses, acute gastroenteritis, zoonoses

## Abstract

We identified a genotype G10P[14] rotavirus strain in 5 children and 1 adult with acute gastroenteritis from the Northern Territory, Australia. Full genome sequence analysis identified an artiodactyl*-*like (bovine, ovine, and camelid) G10-P[14]-I2-R2-C2-M2-A11-N2-T6-E2-H3 genome constellation. This finding suggests artiodactyl-to-human transmission and strengthens the need to continue rotavirus strain surveillance.

Group A rotavirus infection is the major cause of acute gastroenteritis in children worldwide. The rotavirus genome consists of 11 segments of double-stranded RNA encoding 6 structural viral proteins (VP1–4, VP6, VP7) and 6 nonstructural proteins (NSP 1–5/6) (*1*). Genotypes are assigned on the basis of 2 outer capsid proteins into G (VP7) and P (VP4) genotypes; these proteins also elicit type-specific and cross-reactive neutralizing antibody responses (*1*). Strains that include genotypes G1P[8], G2P[4], G3P[8], G4P[8], and G9P[8] cause most rotavirus disease in humans (*1*). Since 2008, rotaviruses have been classified by using the open reading frame of each gene. The nomenclature Gx-P[x]-Ix-Rx-Cx-Mx-Ax-Nx-Tx-Ex-Hx represents the genotypes of the gene segments encoding VP7-VP4-VP6-VP1-VP2-VP3-NSP1-NSP2-NSP3-NSP4-NSP5/6 (*2*). To date, 27 G, 35 P, 16 I, 9 R, 9 C, 8 M, 16 A, 9 N, 12 T, 14 E, and 11 H genotypes have been described (*2*).

Two live oral vaccines are available globally: Rotarix (GlaxoSmithKline Biologicals, Melbourne, Victoria, Australia) and RotaTeq (Merck, Whitehouse Station, NJ, USA). Rotarix is a monovalent vaccine that contains a single human G1P[8] strain (*3*). RotaTeq is a pentavalent vaccine comprised of 5 human–bovine reassortant virus strains (*3*). Both vaccines were introduced into the Australian National Childhood Immunization Program in July 2007. The strategy of a rotavirus vaccination program is to target the most frequently circulating rotavirus strain(s) and provide homotypic and heterotypic protection.

G10P[14] rotavirus strains are rarely reported as the source of infection in humans. Of 7 previously reported G10P[4] rotavirus infections, 1 each was in the United Kingdom and Thailand and 5 were in Slovenia (*4*). During 2011, the Australian Rotavirus Surveillance Program identified 6 G10P[14] strains in the Northern Territory (NT). We report the characterization of G10P[14] strains detected in Australia.

## The Study

Six rotavirus-positive specimens collected from NT were genetically untypeable by reverse transcription PCR (Technical Appendix). Sequence analysis of the VP7 and VP4 genes of these strains demonstrated highest nucleotide identity with G10 and P[14] rotaviruses, respectively. The G10P[14] strains were from specimens collected from 5 children and 1 adult (84 years of age) during August and September 2011 (Table 1). Of the 6 G10P[14] case-patients, 5 were from Tennant Creek, NT, ≈1,000 km south of Darwin in northern Australia; the residence of the other case-patient is unknown. All strains were detected in indigenous Australians. Specimens V585, V582, and WDP280 were collected from case-patients who had received 2 doses of Rotarix, and specimen SA179 was collected from a case-patient who had received 1 dose. No vaccination data were available for the case-patient from whom specimen SA175 was collected.

**Table 1:** Cohort and vaccination status for novel G10P[14] rotavirus strain, Northern Territory, Australia, 2011*

Case-patient specimen ID	Case-patient age	Date specimen collected	Location (postal code) of specimen collection†	Rotavirus vaccine (no. doses)‡
V582	8 mo	Aug 13	0860	Rotarix (2)
WDP280	10 mo	Aug 19	0872	Rotarix (2)
V585	2 y	Aug 19	0860	Rotarix (2)
SA175	3 mo	Sep 2	Unknown	Unknown
SA179	4 mo	Sep 6	0872	Rotarix (1)
D355	84 y	Sep 11	0860	Not applicable

*ID, identification. †Postal code zone 0860 encompasses Tennant Creek, Northern Territory; samples WDP280 and SA179 were collected in post code zone 0872, in communities near code zone 0860. ‡Rotarix, Glaxo SmithKlineBiologicals, Melbourne, Victoria, Australia.

Sanger sequencing was used to generate the complete genome of specimen V585 (Technical Appendix). For the other 5 G10P[14] strains, the complete open reading frames of VP7, VP4, NSP4, and NSP5 and partial reading frames of VP1, VP2, VP3, VP6, NSP1, and NSP2 were sequenced (Table 2, Appendix). These 5 strains demonstrated >99.5% sequence identity to V585, confirming that V585 was representative of all 6 strains. The genotype of each segment of V585 was determined by using RotaC version 2.0 (http://rotac.regatools.be), a web-based genotyping tool for group A rotaviruses; a G10-P[14]-I2-R2-C2-M2-A11-N2-T6-E2-H3 constellation was identified. Maximum-likelihood phylogenetic analyses were performed by using full-length open reading frame nucleotide sequences of V585 and other group A rotavirus strains (Technical Appendix). The nucleotide sequences of the 11 gene segments of V585 and the VP7 and VP4 genes of the other 5 G10P[14] strains were deposited in GenBank (accession nos. JX567748–JX567768).

**Table 2:** Number of nucleotide differences between RVA/human-wt/AUS/V585/2011/G10P[14] and other G10P[14] rotavirus strains*

Strain	RVA/human-wt/AUS/V585/2011/G10P[14] nucleotide sequences†										
VP1 (887)	VP2 (1285)	VP3 (612)	VP4 (2331)	VP6 (1203)	VP7 (981)	NSP1 (1449)	NSP2 (879)	NSP3 (914)	NSP4 (725)	NSP5 (618)
D355	0	0	0	2	0	0	1	0	0	0	0
SA175	1	0	2	2	1	1	1	1	1	1	1
SA179	0	0	0	0	0	0	0	0	1	0	0
V582	0	0	0	0	0	0	0	1	0	1	0
WDP280	0	0	0	0	0	0	0	0	1	0	0
*The number of bases differences between RVA/human-wt/AUS/V585/2011/G10P[14] and other G10P[14] strains for each genome segment is shown. VP, viral protein; NSP, nonstructural protein. †Numbers in parentheses are the final number of nucleotide positions analyzed for each genome segment.											

**Figure 1:**
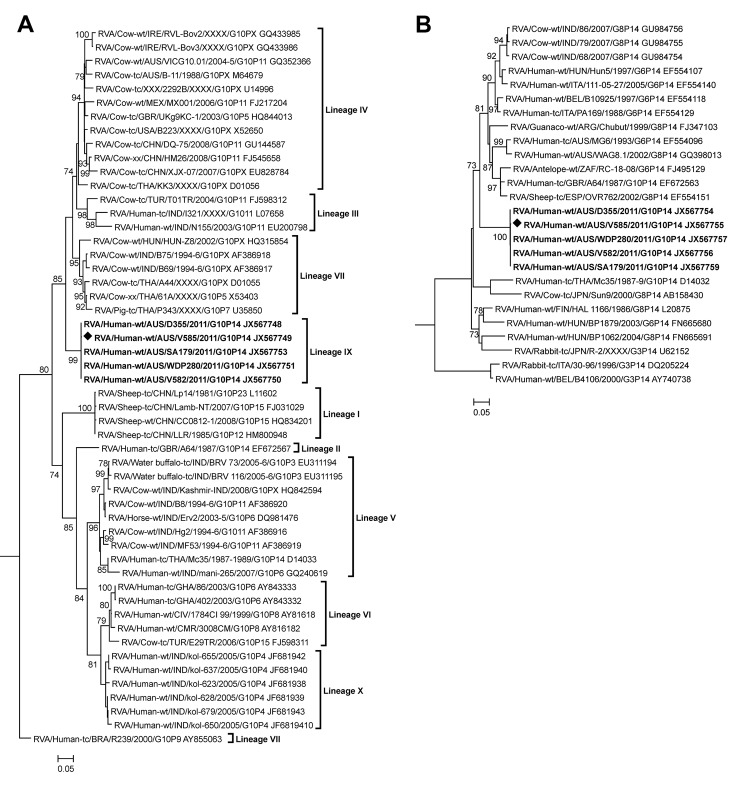
Phylogenetic trees constructed from the nucleotide sequences of viral protein (VP) 7 gene (A) and VP4 gene (B) of rotavirus strain V585; other group A rotavirus strains represent the G10 and P[14] genotypes. The reference strain RVA/human-tc/USA/Wa/1974/G1P[8] was included as an outgroup in the phylogenetic analysis but is not shown in the final tree. The position of strain V585 is indicated by a solid diamond, and all strains from this study are in **boldface**. Bootstrap values >70% are shown. Scale bars show 0.05 nt substitutions per site. The nomenclature of all the rotavirus strains indicates the rotavirus group, species isolated from, country of strain isolation, the common name, year of isolation, and the genotypes for genome segment 9 and 4, as proposed by the Rotavirus Classification Working Group (*2*).

Phylogenetic analysis of the VP7 gene identified 10 lineages (Figure 1, panel A). The 6 G10P[14] strains from NT (lineage IX) were distinct from human G10P[14] rotaviruses RVA/human-tc-GBR/A64/1987/G10P14 (lineage II) and RVA/human-tc/THA/Mc35/1987–1989/G10P[14] (lineage V), and they were most closely related to bovine strains identified predominantly in Ireland, China, and Australia (lineage IV). V585 had the highest level of nucleotide identity (92.5%) to the bovine strain RVA/cow-wt/IRE/RVL-Bov3/XXXX/G10P[X] (Table 3). Nucleotide identity was lower to Australian bovine G10 strains RVA/cow-wt/AUS/VICG10.01/2004-5/G10P[11] (91.1%) and RVA/cow-tc/AUS/B-11/1099/G10P[X] (90.6%).

**Table 3:** Nucleotide identity of 11 genome segments of the G10P[14] rotavirus strain V585, Northern Territory, Australia*

Gene encoding	Genotype of V585	Cutoff value†	Identity of V585 against indicated strains	
			Genotype reference strain	GenBank strains‡
VP1	R2	83	85.6 (DS-1)	96.9 (GirRV)
VP2	C2	84	84.9 (DS-1)	89.5 (B12)
VP3	M2	81	85.1 (DS-1)	88.9 (MG6)
VP4	P[14]	80	87.4 (A64)	88.9 (B10925)
VP6	I2	85	86.6 (DS-1)	93.8 (RotaTeq BrB-9/SC2-9/W17-9)
VP7	G10	80	86.8 (A64)	92.5 (RVL-Bov3)
NSP1	A11	79	79.9 (Hun5)	80.2 (BP1879)
NSP2	N2	85	87.4 (DS-1)	94.3 (B12)
NSP3	T6	85	92.4 (WC3)	94.0 (GirRV/A64)
NSP4	E2	85	88.7 (DS-1)	90.8 (Azuk-1)
NSP5	H3	91	92.9 (AU-1)	94.1 (RUBV81/Egy3399)

*VP, viral structural protein; NSP, nonstructural protein.  †Numeric values are given as percentage nucleotide identity. Percentage nucleotide cutoff values, genotype designation, and genotype reference strains proposed in (*2*).  ‡Strains that shared the highest nucleotide identity with the Australian G10P[14] strain V585.

The VP4 genes of the G10P[14] strains from NT formed a cluster distinct from other characterized P[14] sequences identified globally from humans and animals (Figure 1, panel B). V585 had the highest level of nucleotide identity (88.9%) to the human strain RVA/human-wt/BEL/B10925/1997/G6P[14] (Table 3). Nucleotide identity was lower to other Australian P[14] sequences, RVA/human-tc/AUS/MG6/1993/G6P[14] (87.5%) and RVA/human-wt/AUS/WAG8.1/2002/G8P[14] (87.2%).

**Figure 2:**
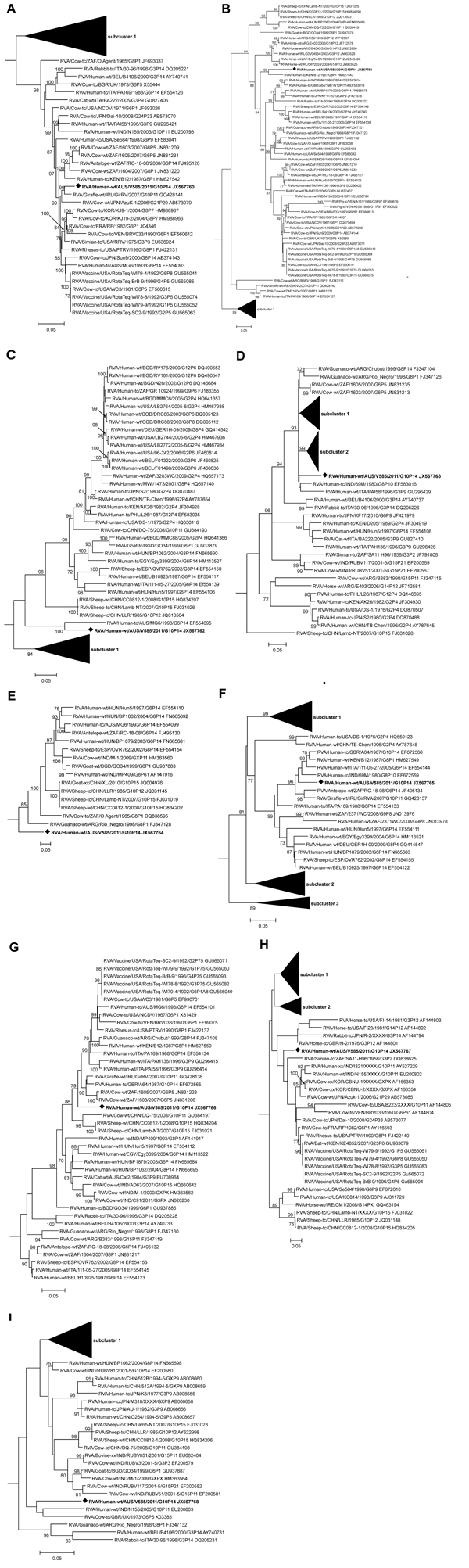
Phylogenetic trees constructed from the nucleotide sequences of genes of rotavirus strain V585 and other group A rotavirus strains representing the R2, C2, M2, I2, A11, N2, T6, E2, and H3 genotypes. A) Viral protein (VP) 1, B) VP2, C) VP3, D) VP6, E) nonstructural protein (NSP) 1, F) NSP2, G) NSP3, H) NSP4, and I) NSP5. The reference strains for each genogroup were included in the phylogenetic analysis, but only the relevant genotype to V585 is shown in the final tree. The position of strain V585 is indicated by an open square, and all strains from this study are in **boldface** font. Bootstrap values >70% are shown. Scale bar shows 0.05 nt substitutions per site. The nomenclature of all the rotavirus strains indicates the rotavirus group, species isolated from, country of strain isolation, the common name, year of isolation, and the genotypes for genome segment 9 and 4, as proposed by the Rotavirus Classification Working Group (*2*).

Phylogenetic analysis of VP1, VP2, NSP2, and NSP3 demonstrated that V585 clustered with genes of rotaviruses identified in the mammalian order Artiodactyla (bovine, ovine, and camelid) and human strains derived from zoonotic infections (Figure 2, Appendix). Similarly, VP3, which clustered with the RVA/human-tc/AUS/MG6/1993/G6P[14], was thought to be the result of zoonotic transmission (*5*) (Figure 2, Appendix, panel C). NSP1, NSP4, and NSP5 clustered with sequences from artiodactyl hosts, however branching was not supported by significant bootstrap values (Figure 2, Appendix, panels E, H, I). The NSP1 and NSP5 genes were divergent from sequences that define their respective genogroups (Table 3). Overall, the 11 genome segments of the G10P[14] strains from NT had relatively low nucleotide identity (80.2%–96.9%) to other strains in each of the respective genogroups, demonstrating that this G10P[14] strain identified in Australia was divergent from other strains identified globally (Table 3).

## Conclusions

The V585 strain possessed a G10-P[14]-I2-R2-C2-M2-A11-N2-T6-E2-H3 genome constellation. With the exception of the VP7 gene, the constellation is consistent with G6P[14] and G8P[14] strains identified globally: G6/G8-P[14]-I2-(R2/R5)-C2-M2-(A3/A11)-N2-T6-(E2/E12)-H3 (*6*). Human P[14] strains are related to rotavirus strains isolated from even-toed ungulates belonging to the mammalian order Artiodactyla (*6*). Consistent with this observation, each individual genome segment of V585 was most closely related to artiodactyl-derived strains or human zoonotic rotavirus strains characterized to be derived from artiodactyl hosts. In Australia, G10P[11] strains have been isolated from calves, and G8P[14] strains and G6P[14] strains have been isolated from children (*7**,**8*). However, the V585 strain demonstrated modest nucleotide identity with these 3 strains identified in Australia. These data suggest that V585 is novel and probably derived from a strain circulating in an artiodactyl host and transmitted to humans. A large feral animal population, including goats, rabbits, and camels, exists in the region where these specimens were collected, thereby supporting the possibility of an interspecies transmission event (*9*).

Vaccination with the monovalent G1P[8] Rotarix is available in NT, where 2-dose vaccine coverage is 74% for indigenous Australian infants (*10*). Rotarix vaccination status was available for 4 of the 5 children in this study: 3 were fully vaccinated, and 1 had received the primary dose. The heterotypic G10P[14] strain identified in these vaccinated children suggests a lack of protective immunity, although it cannot be excluded that vaccination provided protection against severe disease from other genotypes. Vaccine effectiveness against gastroenteritis leading to hospitalization has been variable in NT; vaccine was estimated to be 77.7% effective during a 2007 G9P[8] outbreak (*11*) and 19% effective against a fully heterotypic G2P[4] strain in 2009 (*12*). Rotarix has been effective for decreasing rotavirus infection notification rates in Darwin, NT (*10*), and New South Wales (*13*). However, in 1 location in central NT, reported rotavirus infection rates have remained similar in the vaccine era to those in the prevaccine era (*10*), suggesting low vaccine uptake, low vaccine take, or waning immunity. The living conditions of the indigenous Australian population in central NT are typically crowded, with inadequate facilities for sanitation and food preparation (*14*). The number of diarrheal disease cases is high: admissions coded for enteric infections in NT indigenous Australian infants occur at a rate 10-fold higher than among nonindigenous Australian infants (*15*). The concurrent medical conditions present in the NT indigenous Australian population, combined with diversity of circulating rotavirus types, may have contributed to a lack of immunity. Detection of these unusual G10P[14] strains emphasizes the need for continued rotavirus surveillance to help guide current and future vaccination strategies.

Technical Appendix Detailed descriptions of the methods and materials used and primers used for full genome characterization of rotavirus G10P[14].
