# Supplementary material for: Novel G10P[14] Rotavirus Strain, Northern Territory, Australia
Source: Emerg Infect Dis. 2013 Aug;19(8):1324–7. doi: 10.3201/eid.1908.121653 (PMC3739504; doi:10.3201/eid.1908.121653)
Supplement: Technical Appendix — Detailed descriptions of the methods and materials used and primers used for full genome characterization of rotavirus G10P[14]. [file 12-1653-Techapp-s1.pdf]

Article DOI: http://dx.doi.org/10.3201/eid1908.121653

Novel G10P[14] Rotavirus Strain, Northern Territory, Australia

Technical Appendix

Methods and Materials

Faecal Specimens

Faecal specimens collected from individuals with acute diarrhea were frozen, stored at –70°C and forwarded to the Australian Rotavirus Surveillance Program in Melbourne, Australia. Patient information including age, date of sample collection, sex, immunisation status with regard to the Rotarix rotavirus vaccine and Indigenous status was obtained. The fecal specimens were screened for the presence of other enteric pathogens including ova, cysts, parasites, *Salmonella*, *Shigella*, *Campylobacter*, *Yersinia*, *Vibrio* and shiga-like toxin producing *E. coli* by the pathology services at the site of collection.

Nucleic Acid Extraction

Faecal suspensions (20% wt/vol) were prepared in virus dilution buffer (0.01 mol/L Tris-HCl (pH 7.5), 0.1 mol/L $CaCl_2$, 0.15 mol/L $NaCl_2$). The homogenate was clarified by centrifugation at 16,000 *x* g for 3 min and the supernatant was stored at -20ºC. RNA was extracted from 140 µl fecal suspension using the QIAamp® Viral RNA Mini Kit (QIAGEN, Inc., Hilden, Germany) in accordance with the manufacturer's spin protocol. RNA was eluted in 40 µl RNase-free distilled water and stored at -20 ºC.

G and P Genotyping RT-PCR

All samples were subjected to a two-step hemi-nested multiplex RT-PCR to determine G and P genotype using the One-Step RT-PCR Kit (QIAGEN, Inc., Hilden, Germany), following an established protocol in a PCR thermocycler (96-Well GeneAmp® PCR system 9700, Applied Biosystems). The amplification of the VP7 gene yielded a 881 bp fragment using the oligonucleotide primer set VP7F/VP7R and G genotyping was performed using a pool of primers specific for G1, G2, G3, G4, G8 and G9 (*1-3*). A 663 bp fragment of the VP4 gene was generated using the oligonucleotide primer set VP4F/VP4R and P genotyping was performed using a pool of primers specific for P[4], P[6], P[8], P[9], P[10] and P[11] (*4,5*).

Amplification of VP4 and VP7 Genes

For each of the six G10P[14] samples the full length VP4 and VP7 genes were reverse transcribed and amplified by PCR using the SuperScript III One-Step RT-PCR System with Platinum Taq High Fidelity kit (Invitrogen, USA). Briefly, 5 µl of RNA was denatured at 97°C for 3 min and quenched on ice. Samples were reverse transcribed at 45°C for 30 min, followed by Taq Polymerase activation at 94°C for 2 min, 40 cycles of denaturation at 94°C for 45 sec, annealing at 45°C for 45 sec and extension at 68°C for 5 min were followed by a final extension of 5 min at 68°C. The oligonucleotide primers sets Beg9/End9 (*2*) were used to amplify the VP7 gene and the oligonucleotide primer set VP4 P14 1F/VP4 P14 3R (*6*) were used to amplify the VP4 gene.

Amplification of Nine Remaining Rotavirus Genome Segments

A representative sample of the six G10P[14] strains, V585, was selected for whole genome sequence analysis. RT-PCR was performed under the same conditions outlined above. The oligonucleotide primers used in the amplification of the nine remaining gene segments are listed in Table.

Nucleotide Sequencing

PCR amplicons were excised and purified via gel extraction and spin column purification using the QIAquick Gel extraction Kit (QIAGEN, Inc., Hilden, Germany) according to the manufacturer’s protocol. Purified DNA together with oligonucleotide primers (detailed in Supplementary Table), were sent to the Australian Genome Research Facility, Melbourne and sequenced using an ABI PRISM BigDye Terminator Cycle Sequencing Reaction Kit (Applied Biosystems, Foster City, CA, USA) in an Applied Biosystems 3730xl DNA Analyser (Applied Biosystems, Foster City, CA, USA). Primer walking was employed to cover the complete sequence of each gene.

Phylogenetic Analysis

The electropherograms generated were visually analysed and contiguous DNA sequence files were generated utilising the Sequencher® Software program version 5.0.1 (Gene Codes Corp Inc., Ann Arbor, MI, USA). Nucleotide similarity searches were performed using the BLAST server on the GenBank database at the National Center for Biotechnology Information, USA (www.ncbi.nlm.nih.gov). The deduced nucleotide and amino acid sequences of each gene were compared with sequences available in GenBank that possessed the entire open reading frame (ORF). Multiple nucleotide and amino acid alignments were constructed using ClustalW algorithms in the MEGA5.10 program (*7*). The model of nucleotide substitution

was chosen by following Bayesian information criterion ranking of each alignment as implemented in MEGA5.10. The models selected for each genome segment were Tamura 3-parameter (VP1, VP3, VP7, NSP1, NSP5), Tamura-Nei (VP2, VP4, VP6, NSP4), Hasegawa-Kishino-Yano (NSP2) and General Time Reversible (NSP3). Phylogenetic trees were generated using the Maximum likelihood method and the trees were statistically supported by bootstrap values >70% using 1000 pseudoreplicate runs.

Assignment of Genotypes

In accordance with the recommendations of the Rotavirus Classification Working Group (RCWG), the genotypes of each of the 11 genome segments were determined using the online RotaC v2.0 rotavirus genotyping tool (http://rotac.regatools.be).

Accession Numbers

The nucleotide sequences of the 11 gene segments of V585 and the VP7 and VP4 genes of the other five G10P[14] strains described in this study were deposited in GenBank under the accession numbers JX567748–JX567768.

Technical Appendix Table. Primers used for full genome characterization of G10P[14]

Gene	Primer	Sequence	Binding Position	Reference
VP1	VP1 RV3 Fwd start	GGCTATTAAAGCTATACAATG	1–21	(*8*)
	VP1 P2 R	GGCATCCAACATTTTCTGCT	1045–1025	(*9*)
	VP1 G1 F*	CGAGCNATAGTTCCNGACCA	847–867	(*9*)
	VP1 G5 F*	GCAGCKAATTCAATAGCRAA	1810–1830	(*9*)
	VP1 G6 R*	ATTCGCGCRTAYGTTTCTCT	2003–1984	(*9*)
	CD-VP1-2544 F	TGCACCAATATCACTTGAC	2544–2562	This study
	CD-VP1-2805 R	GCTGATTTTGAACCGTC	2805–2788	This study
	VP1 RV3 Rev end	GGTCACATCTAAGCGCTCTA	3302–3274	(*8*)
VP2	VP2 RV3 Fwd start	GGCTATTRAAGGYTCAATGG	1–20	(*8*)
	VP2 P2 R	AACAAAATGYAGCCAATTC	1441–1423	(*9*)
	VP2 G1 F	TCATTAATTTCAGGYATGTGG	1223–1244	(*9*)
	CD-VP2-2100R	GCACGTTCTATYTGATCC	2100–2083	This study
	DC-VP2-585R	TCCAATACCATCTGTTACGC	382–402	This study
	DC-VP2-585F	ATAACTTTGAGTCGCTGTGG	892–911	This study
	DC-VP2-585F	AGAATTAATGCGGACAGGCG	2165–2184	This study
	VP2 RV3 Rev end	GGTCATATCTCCACARTGG	2687–2669	(*8*)
VP3	CD-G10P14-VP3-23F	GTGTGTTTTACCTCTGATGG	23–43	This study
	VP3 RV3 Rev end	GGYCACATCATGACTAGTG	2591–2572	(*8*)
	CD-G10P14-VP3-917F	ATCAGCACCATCGTACTGGA	917–937	This study
	CD-G10P14-VP3-509R	CATCATCATCCGTAGCCG	509–491	This study
	CD-G10P14-VP3-1776R	CGTATTTGATGCGACCGT	1776–1758	This study
	CD-G10P14-VP3-2470F	CAGTGAGAATAGATACCACG	2470–2490	This study
VP4	VP4 P14 1F	GGCTATAAAATGGCTTCTTT	1–20	(*6*)
	VP4 P14 2R	AAAGATGGTTCACTAACAGC	1320–1300	(*6*)
	CD VP4 P14 1191F	CCAGTAATGAGTGGTGGC	1191–1208	This study
	VP4 P14 3R	GGTCACATCYTWARCAGACAG	2361–2341	(*6*)
	DC-VP4-585R	GAGCATACCCAGTCTGTGC	116–134	This study
	DC-VP4-585F	TACACTGCCGACAGATTTCG	988–1007	This study
	VP4 P14 1R	CTTCATTCATTCATTTGTGC	771–751	(*6*)
	VP4 P14 3F	ATGTCAGATGCGGCTTCTTC	1711–1730	(*6*)
VP6	VP6 F	GGCTTTWAAACGAAGAAGTCTT	1–19	(*8*)
	VP6 R	GGTCACATCCTCTCACT	1356–1340	(*8*)
	CD-G10P14-VP6-700F	CCAGATGCAGAAAGATTTAG	701–721	This study

Gene	Primer	Sequence	Binding Position	Reference
	CD-G10P14-VP6-521R	CAGTGTAAATGACGCTGAA	521–502	This study
VP7	Beg9	GGCTTTAAAAGAGAGAATTTCCGTCTGG	1–23	(*2*)
	End9	GGTCACATCATACAATTCTAATCTAAG	1062–1035	(*2*)
	DC-VP7 585 R	TGATACATCCATCGATCCAG	183–202	This study
	DC-VP7 585 F	ATCTGATCCAACGACTGCTC	820–839	This study
NSP1	NSP1_5'UTR	TGTAAAACGACGGCCAGTTATGAAAAGTCTT GTGGAAGC		JCVI
	NSP1_3'UTR	CAGGAAACAGCTATGACCCATTTTATGCTGC CTAGGCG		JCVI
	CD-G10P14-NSP1-1180F	ATGAAGAACGGTGGACTGAG	1180–1120	This study
	CD-G10P14-NSP1-695F	GGAATTGGTAAGAAACGAC	695–714	This study
	CD-G10P14-NSP1-531R	CCTGCTCCATAAAATCATAG	531–511	This study
NSP2	NSP2F	GGCTTTTAAAGCGTCTCAG	1–19	(*8*)
	NSP2R	GGTCACATAAGCGCTTTC	1059–1042	(*8*)
	DC-NSP2-585R	TAAATTGTGGCGGTGGTGC	152–170	This study
	DC-NSP2-585F	ATGAAGCGGGAGAGTAATCC	874–894	This study
NSP3	NSP3F	GGCTTTTAATGCTTTTCAGTG	1–21	(*8*)
	NSP3R	ACA TAA CGC CCC TAT AGC	1070–1054	(*8*)
	CD-G10P14-NSP3-923F	AGAGGACTAACGAAGCAATG	923–943	This study
NSP4	10.1	GGCTTTTAAAAGTTCTGTCC	1–20	Not previously published
	10.2	GGTCACACTAAGACCATTCC	750–730	Not previously published
NSP5	NSP5F	GGCTTTTAAAGCGCTACAG	1 – 19	(*8*)
	NSP5R	GGTCACAAAACGGGAGT	667–647	(*8*)

*Primer with degenerate sites inserted.

References

1. Iturriza-Gomara M, Cubitt D, Desselberger U, Gray J. Amino acid substitution within the VP7 protein of G2 rotavirus strains associated with failure to serotype. J Clin Microbiol. 2001;39:3796–8. PubMed http://dx.doi.org/10.1128/JCM.39.10.3796-3798.2001

2. Gouvea V, Glass RI, Woods P, Taniguchi K, Clark HF, Forrester B, et al. Polymerase chain reaction amplification and typing of rotavirus nucleic acid from stool specimens. J Clin Microbiol. 1990;28:276–82. PubMed

3. Iturriza-Gomara M, Kang G, Gray J. Rotavirus genotyping: keeping up with an evolving population of human rotaviruses. J Clin Virol. 2004;31:259–65. PubMed http://dx.doi.org/10.1016/j.jcv.2004.04.009

4. Simmonds MK, Armah G, Asmah R, Banerjee I, Damanka S, Esona M, et al. New oligonucleotide primers for P-typing of rotavirus strains: Strategies for typing previously untypeable strains. J Clin Virol. 2008;42:368–73. PubMed http://dx.doi.org/10.1016/j.jcv.2008.02.011

5. Gentsch JR, Glass RI, Woods P, Gouvea V, Gorziglia M, Flores J, et al. Identification of group A rotavirus gene 4 types by polymerase chain reaction. J Clin Microbiol. 1992;30:1365–73. PubMed

6. Fukai K, Saito T, Inoue K, Sato M. Molecular characterization of novel P[14],G8 bovine group A rotavirus, Sun9, isolated in Japan. Virus Res. 2004;105:101–6. PubMed http://dx.doi.org/10.1016/j.virusres.2004.04.016

7. Tamura K, Peterson D, Peterson N, Stecher G, Nei M, Kumar S. MEGA5: molecular evolutionary genetics analysis using maximum likelihood, evolutionary distance, and maximum parsimony methods. Mol Biol Evol. 2011;28:2731–9. PubMed http://dx.doi.org/10.1093/molbev/msr121

8. Rippinger CM, Patton JT, McDonald SM. Complete genome sequence analysis of candidate human rotavirus vaccine strains RV3 and 116E. Virology. 2010;405:201–13. PubMed http://dx.doi.org/10.1016/j.virol.2010.06.005

9. Ramani S, Iturriza-Gomara M, Jana AK, Kuruvilla KA, Gray JJ, Brown DW, et al. Whole genome characterization of reassortant G10P[11] strain (N155) from a neonate with symptomatic rotavirus infection: identification of genes of human and animal rotavirus origin. J Clin Virol. 2009;45:237–44. PubMed http://dx.doi.org/10.1016/j.jcv.2009.05.003
